# *Sulawesifulvius thailandicus* – a new species of the genus *Sulawesifulvius* Gorczyca, Chérot & Štys from Thailand (Hemiptera, Heteroptera, Miridae, Cylapinae)

**DOI:** 10.3897/zookeys.647.10960

**Published:** 2017-01-26

**Authors:** Andrzej Wolski, Tomohide Yasunaga, Jacek Gorczyca, Aleksander Herczek

**Affiliations:** 1 Department of Biosystematics, Opole University, Oleska 22, 45-052 Opole, Poland; 2 Research Associate, Division of Invertebrate Zoology, American Museum of Natural History, New York c/o Nameshi 2-33-2, Nagasaki 852-8061, Japan; 3 Department of Zoology, University of Silesia, Bankowa 9, 40-007 Katowice, Poland

**Keywords:** Miridae, Cylapinae, Sulawesifulvius, description, diagnosis, Oriental Region

## Abstract

A new species of the genus *Sulawesifulvius*, *Sulawesifulvius thailandicus* Wolski, Yasunaga & Gorczyca, **sp. n.**, is described from Thailand. The present finding also represents the first distribution record in Indochina for the genus. Color adult habitus images for *Sulawesifulvius thailandicus* and *Sulawesifulvius schuhi* (type species of the genus), male genital drawings of *Sulawesifulvius thailandicus*, and scanning electron micrographs of selected structures of *Sulawesifulvius schuhi* and *Sulawesifulvius thailandicus* are provided.

## Introduction

*Sulawesifulvius* Gorczyca, Chérot & Štys, 2004 is a unique cylapine genus established by Gorczyca et al. (2004) to accommodate a single species, *Sulawesifulvius schuhi* Gorczyca, Chérot & Štys, 2004, described from Sulawesi, Indonesia. Recently, Mu and Liu (2014) added the new species *Sulawesifulvius yinggelingensis* Mu & Liu from Hainan (China) and Yeshwanth and Chérot (2015) described a new species, *Sulawesifulvius indicus* Yeshwanth & Chérot, from India, which significantly expanded the distribution range of *Sulawesifulvius* from the Wallacea to the Oriental Region (Fig. 22).

In this paper, a new species, *Sulawesifulvius thailandicus*, is diagnosed and described based on material recently collected in central Thailand. Habitus photographic images of *Sulawesifulvius schuhi* (type species of the genus) and *Sulawesifulvius thailandicus* sp. n., male genitalic drawings of *Sulawesifulvius thailandicus* sp. n., and scanning electron micrographs of the selected structures of *Sulawesifulvius schuhi* and *Sulawesifulvius thailandicus* are provided. The present discovery of a new species in Thailand also represents the first distributional record from Indochina for the genus.

## Materials and methods

Observations were made using an Olympus SZX12 stereomicroscope and an Olympus BX50 optical microscope. Digital images of live individuals were taken by TY with Canon EOS Kiss digital camera body + Olympus OM-System. Scanning electron micrographs were taken using Hitachi S-3400N and Phenom XL Scanning Electron Microscopes. Measurements were taken using an eyepiece (ocular) micrometer; all measurements are given in millimeters.

Dissections of male genitalia were performed using the technique mentioned by Kerzhner and Konstantinov (1999). The terminology of the male genitalic structures follows Konstantinov (2003) for the elements of the genital capsule and parameres and Cassis (2008) in using the term “endosoma” for the male intromittent organ. The study was based on the material deposited in the Insect Collection, Entomology & Zoology Group, Plant Protection Research and Development Office, Department of Agriculture, Bangkok (DOA), T. Yasunaga Collection, Nagasaki, Japan (TYCN); and Department of Zoology, University of Silesia, Poland (US).

## Taxonomy

### Sulawesifulvius thailandicus

Taxon classificationAnimaliaHemipteraMiridae

Wolski, Yasunaga & Gorczyca sp. n.

http://zoobank.org/CCBBF52D-B8B6-4418-A2F1-B782B3A5F5D8

Figures 2–4 , 12–17 , 18–21 , 22–25

#### Diagnosis.

Recognized by the following set of characters: dorsum yellow with large dark brown and red areas (Figs 2–3); parameres as described below and depicted in Figs 14–17; endosoma with three well-developed sclerites (Fig. 13).

**Figures 1–4.**
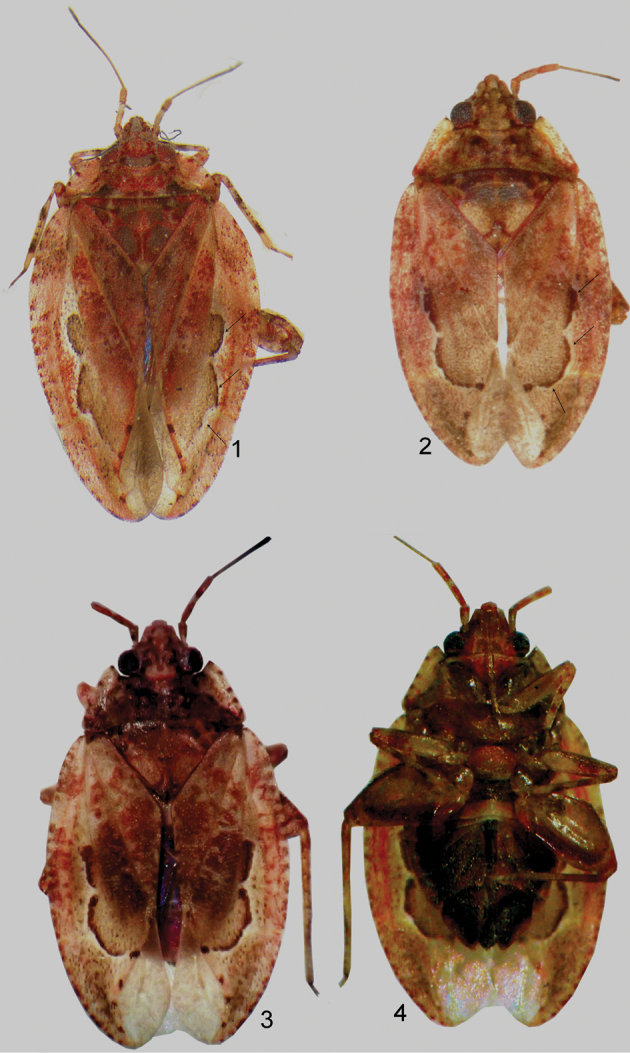
Dorsal habitus photograph of *Sulawesifulvius schuhi* (**1** ♂ paratype) and *Sulawesifulvius thailandicus* (**2** holotype **3** ♀ paratype). Ventral view of *Sulawesifulvius thailandicus* (**4** ♀ paratype).

**Figures 5–11.**
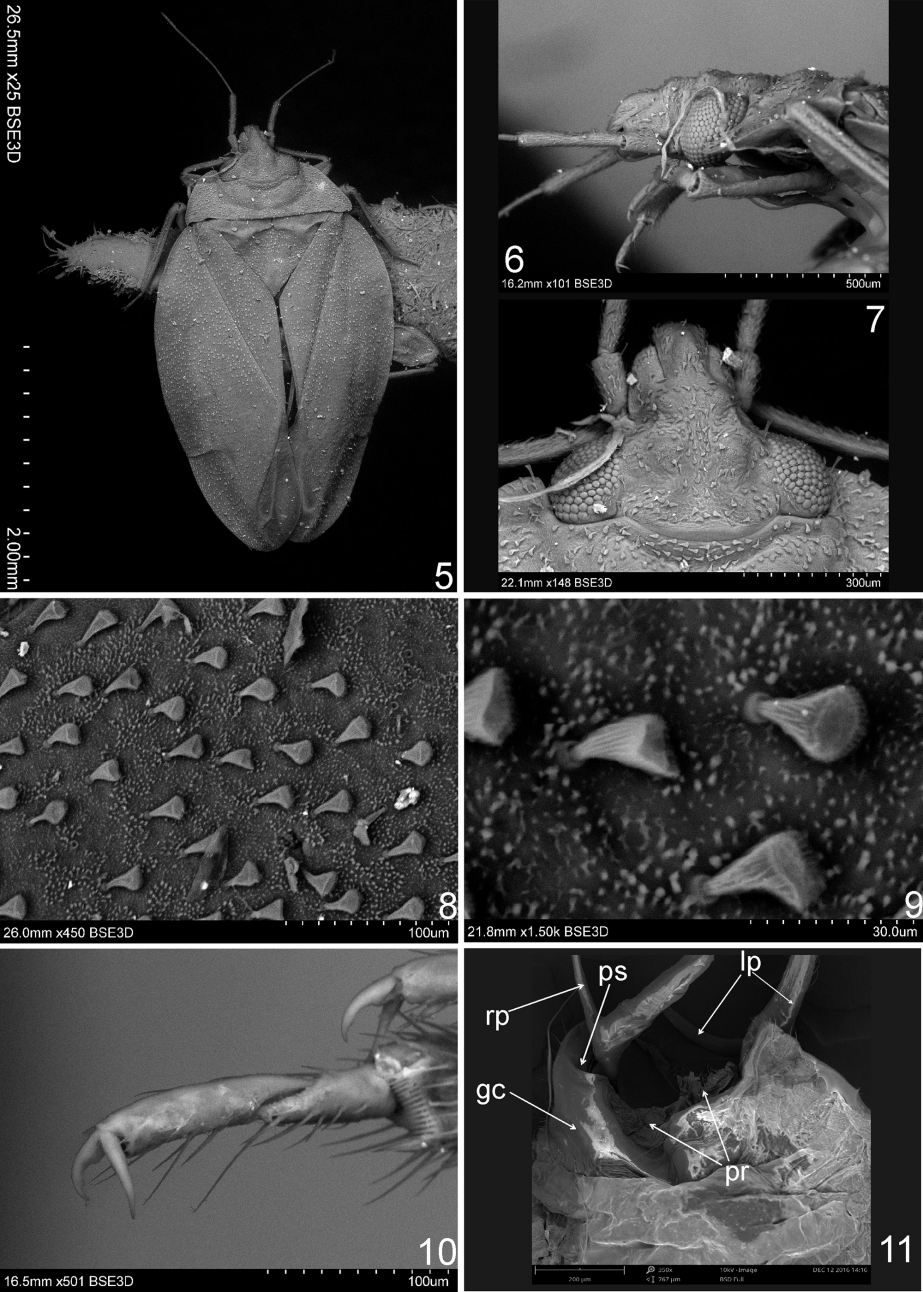
Scanning electron micrographs of *Sulawesifulvius schuhi* (♂, paratype). **5** Dorsal habitus **6** Head and pronotum (lateral view) **7** Head (dorsal view) **8, 9** Structure and vestiture of hemelytron **10** Protarsus **11** Posterior half of male abdomen (dorsal view). Abbreviations: gc = genital capsule; lp = left paramere; pr = proctiger; ps = paramere socket; rp = right paramere.

**Figures 12–17.**
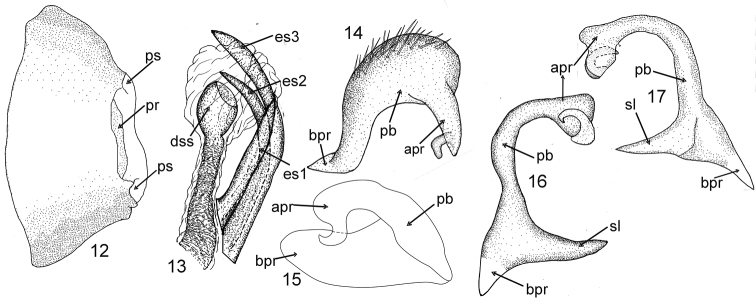
Male genitalia of *Sulawesifulvius thailandicus*. **12** Genital capsule (dorsal view, aedeagus and parameres removed) **13** Endosoma (right lateral view) **14** Right paramere (right lateral view) **15** Right paramere (dorsal view) **16** Left paramere (right lateral view) **17** Left paramere (let lateral view). Abbreviations: apr = apical process; bpr = basal process; dss = sclerotized portion of ductus seminis inside endosoma; es 1, 2, 3 – endosomal sclerites; pb = paramere body; pr = proctiger; ps = paramere socket; sl = sensory lobe.

*Sulawesifulvius thailandicus* is most similar to *Sulawesifulvius schuhi* and *Sulawesifulvius yinggelingensis* in having large red markings on the dorsal surface (Figs 2–3, 20, 23; Mu and Liu 2014) (only slightly tinged with red in *Sulawesifulvius indicus*) (Yeshwanth and Chérot 2015). *Sulawesifulvius thailandicus* can, however, be easily distinguished by the characteristic shape of the parameres (as depicted in Figs 14–17) and the endosoma with three endosomal sclerites (Fig. 13).

**Figures 18–21.**
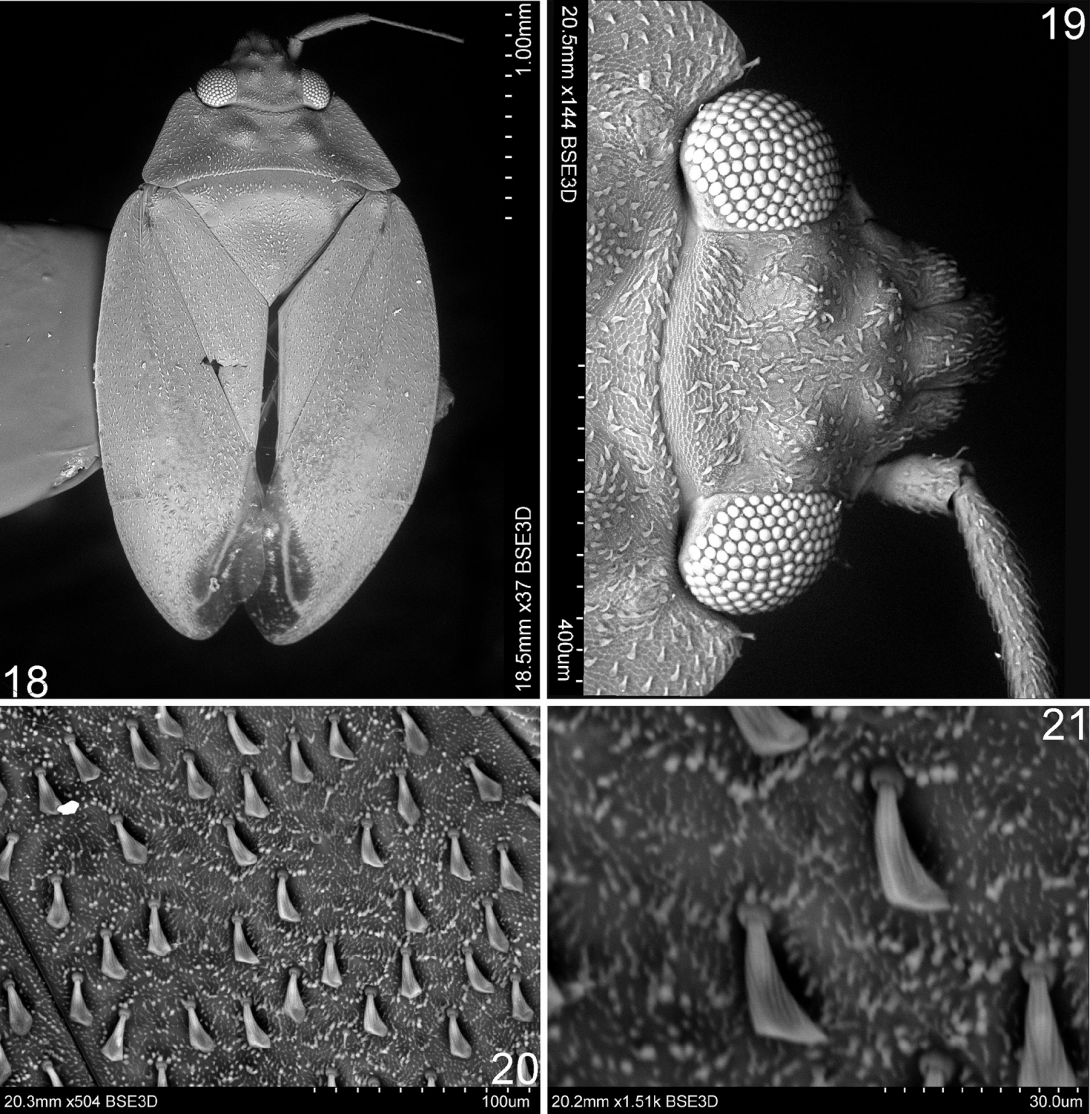
Scanning electron micrographs of *Sulawesifulvius thailandicus* (holotype) **18** Dorsal habitus **19** Head (dorsal view) **20–21** Structure and vestiture of hemelytron.

**Figures 22–25.**
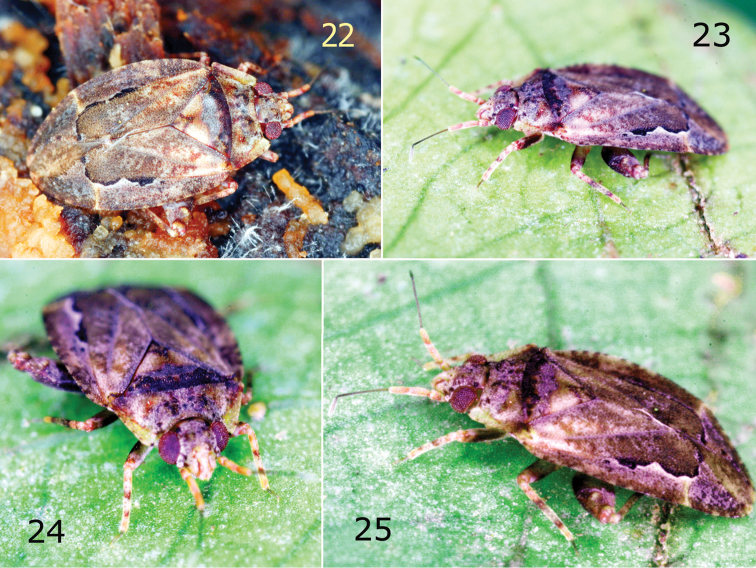
*Sulawesifulvius thailandicus*, a male adult live individual (SERS).

#### Description.

**Coloration** (Figs 2–4, 22–25). Dorsum yellow extensively mottled with dark brown and red. ***Head***. Ground coloration yellow; vertex and frons moderately tinged with brown; vertex with two blackish patch posteriorly, each bordering inner margin of eye; tubercles on vertex and frons each with small dark brown patch; mandibular and maxillary plates and clypeus tinged with red; mandibular plates with two small, dark brown patches basally; antennal segments I and II yellow broadly tinged with red; segment dark brownish; labium dirty yellow tinged with red. ***Thorax***. *Pronotum*. Yellow broadly tinged with brown and red; calli tinged with black. *Mesoscutum and scutellum*. Mesoscutum dark brown with small yellow tinges; scutellum mostly yellow, dark brown basally, with brown triangular pattern apically and with small, brown, longitudinal patch at extreme apex. *Thoracic pleura*. Proepimeron yellowish, weakly tinged with red; mesepimeron and metepisternum dark castaneous; scent gland evaporative area dirty yellow. *Hemelytron*. Yellow, extensively tinged with dark brown, brown, and red; apical half of exocorium and basal portion of cuneus with marking composed of black, longitudinal, curved patches bordering R+M vein and inner half of basal margin of cuneus (Fig. 2, arrow); membrane fuscous mottled with yellow. *Legs*. Dirty yellow with dark brown, brown and red tinges. ***Abdomen***. Dark brown. **Structure, Texture, and Vestiture** (Figs 2–4, 18–25). ***Head***. Vertex and frons each with pair of relatively large tubercles; labial segments I and II subdivided. *Pronotum*. Calli small. *Mesoscutum and scutellum*. Scutellum flat.

***Male genitalia*** (Figs 12–17). *Genital capsule* (Fig. 12). Weakly flattened dorsoventrally; dorsal wall long, only weakly shorter than ventral wall; proctiger narrow; genital opening terminal in orientation; lateral margin immarginate. *Aedeagus* (Fig. 13). *Ductus seminis* relatively broad and rather short; sclerotized part of ductus seminis inside endosoma (dss) ovoid; secondary gonopore clearly present; endosoma with three sclerites (es1, es2 and es3); each sclerite sharply pointed; es1 nearly cylindrical at basal three fourths, apical one fourth tapering toward apex; sclerites es2 and es3 strongly curved; es2 tapering toward apex; es3 weakly broadened apically. *Right paramere* (Figs 14–15). Apical process relatively short, tapering toward apex, with protruding, hook-shaped process subapically; paramere body broad, covered with relatively long, semierect setae dorsally; basal process triangular. *Left paramere* (Figs 16–17). Apical process strongly broadened toward apex, with protruding hook-shaped process apically; paramere body long and thin, its lateral margins sinuate; sensory lobe and basal process strongly developed, elongated, tapering toward apex and sharply pointed

**Measurements.** ♂ (*: holotype measurements): Body length 2.95–3.05*, width 1.6–1.75*; Head length 0.48–0.53*, width across eyes 0.65–0.68*, interocular distance 0.3–0.33*; antennal segments I 0.14*–0.16, II 0.37–0.40*, III 0.46*–0.53, IV 0.17; labium obscured by glue and immeasurable in the examined specimens); pronotal length 0.53–0.55*; anterior margin 0.73–0.75*, lateral margin 0.3, posterior margin 1.26–1.33*. ♀: Body length 3.45, width 1.95; Head length 0.57, width across eyes 0.72, interocular distance 0.33; antennal segments I 0.15, II 0.45, III 0.57, IV missing; labial length 1.05; pronotal length 0.55, anterior margin 0.82, lateral margins 0.60, posterior margin 1.35.

**Female.** Similar to male in coloration, structure, texture, and vestiture.

#### Biology.

Unknown.

#### Distribution.

Thailand (Nakhon Ratchasima, Nakhon Nayok).

**Figure 26.**
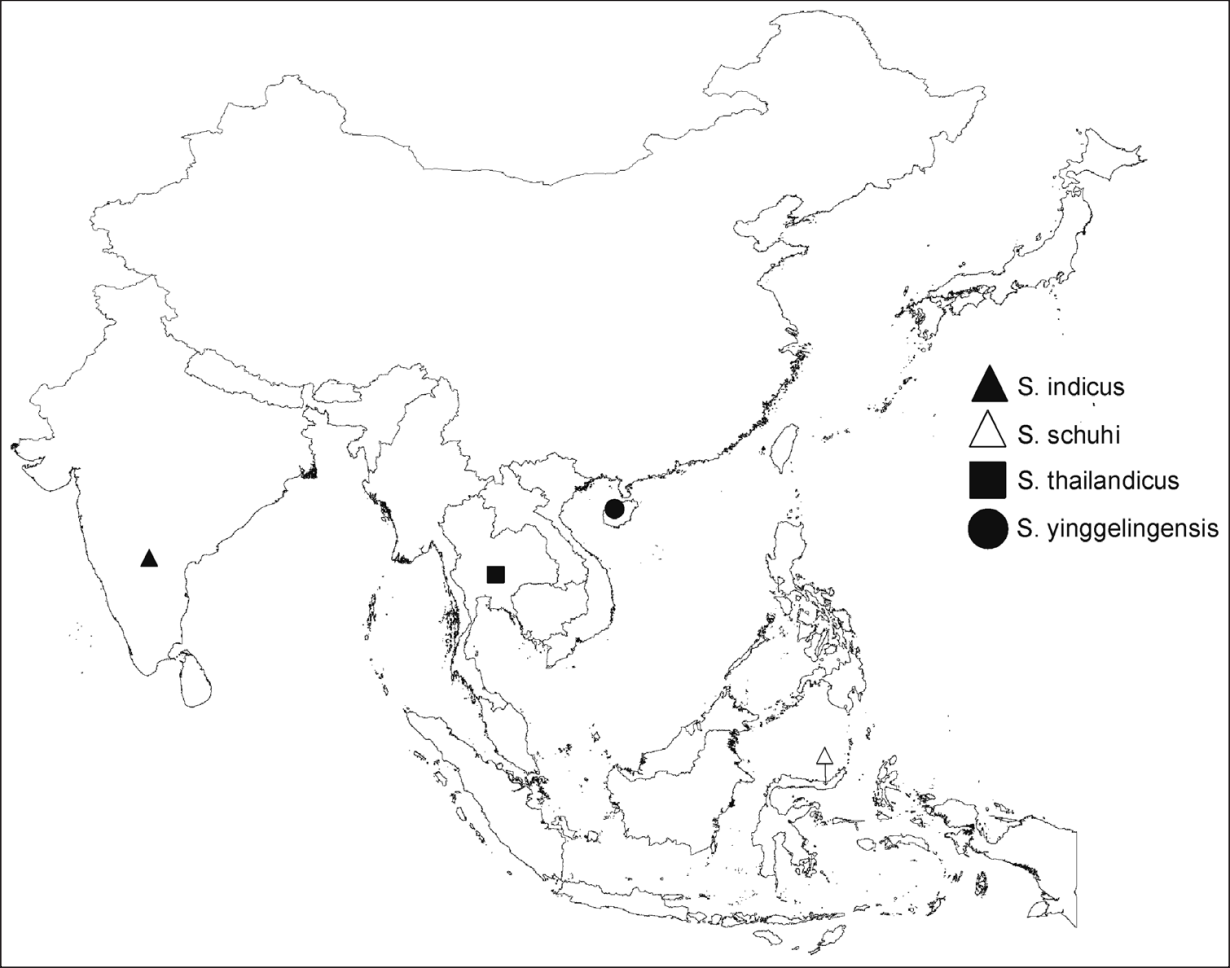
Distribution map of *Sulawesifulvius*.

#### Etymology.

The specific name refers to the country where the holotype was collected.

#### Type material.

**Holotype male**. Nakhon Ratchasima Prov., Wang Nam Khieo, Sakaerat Environmental Research Station, Sakaerat Biosphere Reserve, 14°30'27"N, 101°55'39"E, 410 m alt., light trap, 25 Sep 2013, T. Yasunaga (**DOA**); **paratypes** ♂: Nakhon Ratchasima Prov., Wang Nam Khieo, Sakaerat Environmental Research Station, Sakaerat Biosphere Reserve, 14°30'27"N, 101°55'39"E, *Dipterocarpus* forest, light trap, 22 July 2015, leg. J. Gorczyca & A. Herczek (**US**); ♀: THAILAND: Nakhon Nayok: Sarika, N14°18'39" E101°18'00", at light, 22 Mar 2010, T. Yasunaga & K. Yamada (AMNH_PBI 00380553) (**TYCN**).

## Discussion

Gorczyca et al. (2004) included *Sulawesifulvius* in the subfamily Cylapinae, based on the presence of the two-segmented tarsus and the claw with a subapical tooth (Fig. 10). The placement of *Sulawesifulvius* in the tribe Fulviini *sensu* Gorczyca (2000, 2006) was based on its horizontally elongated head and short antenna (Figs 1–7, 18–19, 22–24). Our examination revealed that *Sulawesifulvius* possesses the subdivided labial segment I which is shared by most genera currently placed in Fulviini (Wolski and Henry 2012, 2015; Wolski 2013; Wolski and Gorczyca 2014) but is not present in other tribes of Cylapinae, except for such rhinomirines as *Rhinomiris* Kirkaldy (Wolski and Henry 2015), *Lundbladiola* Carvalho, *Pararhinomiris* Gorczyca, *Rhinomiridius* Poppius, and *Rhinomiriella* Gorczyca (Wolski, pers. obs.). Other characters that warrants a placement of *Sulawesifulvius* within the tribe Fulviini include among others: 1) the dorsal surface with ornamentation composed of dense, tiny tubercles (Figs 7–8; 19–21); 2) the labial segment II subdivided subapically; 3) eyes with dense interocular setae (Figs 7, 19); 4) the metathoracic gland evaporative area narrowly developed, restricted to ventral margin of metepisternum; 5) the two segmented tarsus (Fig. 10); 6) the genital capsule with the dorsal wall long, only weakly shorter than ventral wall, genital opening terminal in orientation (Fig. 12; Gorczyca et al. 2004: fig. 6; Mu and Liu 2014: fig. 5; Yeshwanth and Chérot 2015: fig. 1).

Similar, shagreened surface of the dorsum to that found in *Sulawesifulvius* is present in many genera of Fulviini (Gorczyca and Wolski 2007: fig. 9; Wolski and Henry 2012: Figs 27–32, 54, 58, 60–61, 73–74; Pluot-Sigwalt and Chérot 2013: Figs 2A–B; Wolski and Gorczyca 2014: fig. 29–31) and is not found in other tribes of Cylapinae except for some rhinomirines (Wolski, pers. obs.).

The subdivision of the labial segment II in the Fulviini was noted among others by van Doesburg (1985: fig. 5) and Namyatova et al. (2016: fig. 10A). This character was also noted for *Psallops* Usinger and was not found in the representatives of the remaining cylapine tribes (Namyatova et al. 2016).

In *Sulawesifulvius* the dorsal wall of the genital capsule is long, weakly shorter than ventral wall and the genital opening is terminal in orientation (Fig. 12). Similar shape of the genital capsule was noted for the fulviine genera *Peritropis* (Moulds and Cassis 2006: fig. 1H; Yeshwanth et al. 2016: Figs 34, 39, 45, 52, 57, 62), *Mimofulviella* Wolski (Wolski 2008), *Euchilofulvius* Poppius (Yeshwanth et al. 2016: fig. 10) and *Fulvius* Stål (Yeshwanth et al. 2016: Figs 16, 21, 34, 39) and is very common among other fulviine genera (Wolski, pers. obs.). In other cylapine tribes the dorsal wall of the genital capsule is shorter than the ventral wall and the genital opening is directed more upwards (Yasunaga 2000: fig. 5; Cassis et al. 2003: Figs 1H, 2E; Cassis and Monteith 2006: fig. 3A; Wolski 2010: Figs 6A, 15A; Wolski and Gorczyca 2012: Figs 79, 83; Yeshwanth et al. 2016: Figs 2, 76).

*Sulawesifulvius* was diagnosed by Gorczyca et al. (2004), Mu and Liu (2014), and Yeshwanth et al. (2016). The most distinctive characters of *Sulawesifulvius*, not shared by any other known fulviines, include the antennal segment III longest (Figs 1–3), the cuneus long, curved, nearly enveloping membrane (Figs 1–3, 5, 18, 22, 25), and the enlarged metafemur with subapical depressions laterally (Gorczyca et al. 2004: fig. 2). An additional character that could clearly distinguish *Sulawesifulvius* from other Fulviini is the characteristic marking on the hemelytron composed of blackish, longitudinal, curved patches occupying apical half of the exocorium, bordering R+M vein and inner half of the basal margin of the cuneus (Figs 1, 2 (arrows); Mu and Liu 2014: 1–2; Yeshwanth and Chérot 2015: Figs 8–9). The shape of the parameres in *Sulawesifulvius* species (except for *Sulawesifulvius indicus*) is bizarre, not exhibited by any other fulviine (Figs 14–17; Gorczyca et al. 2004: 6–9, Mu and Liu 2014: 7–10). By the oval body (Figs 1–3, 5, 18, 22) and the short pronotum with elevated lateral margins and anterior angle protruding onto eyes (Figs 1–5, 7, 18–19, 22) *Sulawesifulvius* is most similar to the genus *Peritropis* Uhler, from which it can be easily distinguished by the characters mentioned above.

## Supplementary Material

XML Treatment for Sulawesifulvius thailandicus
